# Intraductal papillary mucinous neoplasm complicated with intraductal bleeding in a young woman mimicked a cystic solid pseudo-papillary tumor: a case report

**DOI:** 10.1186/s12876-020-01436-3

**Published:** 2020-09-16

**Authors:** Jianman Wu, Yin Lin, Jingwen Wu

**Affiliations:** 1 Department of Radiology, Fujian Provincial Hospital, Provincial clinic medical college of Fujian Medical University, NO.134, Eastern Road, Gulou District, Fuzhou, China; 2 Department of Pathology, Fujian Provincial Hospital, Provincial clinic medical college of Fujian Medical University, NO.134, Eastern Road, Gulou District, Fuzhou, China; 3 Department of Radiology, Fujian Provincial Hospital, Provincial clinic medical college of Fujian Medical University, NO.134, Eastern Road, Gulou District, Fuzhou, China

**Keywords:** Pancreas, Intraductal papillary mucinous neoplasm, Hemorrhage, Computed tomography, Magnetic resonance imaging

## Abstract

**Background:**

There are only 6 cases of intraductal papillary mucinous neoplasm (IPMN) complicated with intraductal hemorrhage have been reported in English literatures. All these 6 cases of IPMN occurred in the old people. The present rare case of IPMN complicated with intraductal hemorrhage occurred in a young woman, and mimicked a cystic solid pseudo-papillary neoplasm (SPN) on preoperative imaging findings.

**Case presentation:**

A 29-year-old young woman complained of a sustained mild right upper quadrant abdominal pain. CT and MRI showed a lobulated, partly ill-defined cystic lesion located in the pancreatic head. Spotted calcification within cystic wall was seen on CT. The lesion was demonstrated as predominantly homogeneous hyperattenuation on CT and homogeneous high signal without decreased signal on fat suppression sequence on T1WI. After contrast administration, the cystic wall and septa of lesion was showed gradually mild to moderate degree of enhancement over time both on CT and MRI. No communication between lesion and the main duct was found on MRCP and the main pancreatic duct and common bile duct were not dilated. Considering patient’s age, gender and manifestations of lesion on CT and MRI (calcification, bleeding and gradually enhanced pattern), the present case mimicked as a cystic SPN. The lesion was pathologically confirmed a branch type IPMN after surgical resection.

**Conclusion:**

We propose that IPMN may need to be taken into account in the differential diagnosis when pancreatic cystic lesions occur in young women with bleeding, calcification, progressive enhancement of cystic wall and no communication with the main pancreatic duct.

## Background

Intraductal papillary mucinous neoplasm (IPMN) complicated with intraductal hemorrhage is rarely seen. To the best of our knowledge, there were only 6 cases of IPMN complicated with bleeding have been reported in English literatures and all of them occurred in the elder people (average age, 70, range, 60–77) with a male gender predisposition (4 males, 2 females) [1, 2] (Table 1).

**Table 1:** Summery of reported IPMN with complication of intraductal bleeding according to English literatures

	Sex	Age	Site	Size (cm)	symptom	Tumor makers /Amylase	CT and /or MR	calcification	Volume of Bleeding	Main duct dilation	Subtype	Degree of dysplasia
Yamada et al. [1]	M	65	Head	N.D.	Abdominal pain	Normal/Normal	CT	none	little	no	BD	IPMA
	M	60	Tail	N.D.	Abdominal pain	Normal/Normal	CT and MR	none	little	yes	BD	IPMA
	F	73	Head	N.D.	none	CEA elevated/Normal	CT and MR	none	little	no	BD	IPMC
	F	77	Body-Tail	N.D.	none	Normal/Amylase elevated	CT and MR	none	little	yes	MD	IPMA
	M	71	Head-Tail	N.D.	none	CA19-9elevated/Normal	CT and MR	none	little	yes	MD	Invasive IPMN high-grade dysplasia
Tokue H [2].	F	74	Head	5.5	Abdominal pain	Normal/Normal	CT	none	Massive in abdominal cavity	no	BD	Low-grade dysplasia
Present case	F	29	Head	5.7	Abdominal pain	Normal/Normal	CT and MR	Spotted	Filled	no	BD	Mild dysplasia

*N.D.* not detailed, *BD* branch-duct, *MT* mixed-type, *MD* main-duct, *IPMA* intraductal papillary mucinous adenoma, *IPMC* intraductal papillary mucinous carcinoma

Here, we present a rare case of IPMN complicated with bleeding in a 29-years-old young woman, and mimicked a cystic solid pseudo-papillary neoplasm (SPN) on preoperative imaging findings.

## Case presentation

A 29-year-old young woman, who was 4 months later the end of her first pregnancy and during lactation, complained of a persistent mild pain in her right upper quadrant abdomen for 3 days. No fever, jaundice and weight loss. She had history of cholecystolithiasis, denied history of biliary colics and acute pancreatitis. She was Hepatitis B virus carrier, denied hypertension/pregnancy hypertension and history of diabetes, No surgery and injury history. No smoking and history of alcohol and drug use. Her menarche age was 13 years old, last menstrual period (LMP): 2015.07.05. Her marriage age was 28 years old. Natural birth of her first child. She denied the history of hypertension, diabetes, and tumors.

Her Body Mass Index (BMI) was 26.3 (height, 163 cm; weight, 70 kg), but her waist circumference was not obtained. Physical examinations suspected positive Murphy sign. Laboratory examinations results showed white blood cell count and amylase was normal. Tumor makers of carbohydrate antigen 19–9 (CA199), carcinoembryonic antigen (CEA), alpha-fetoprotein (AFP) were within the normal limits.

**Fig. 1:**
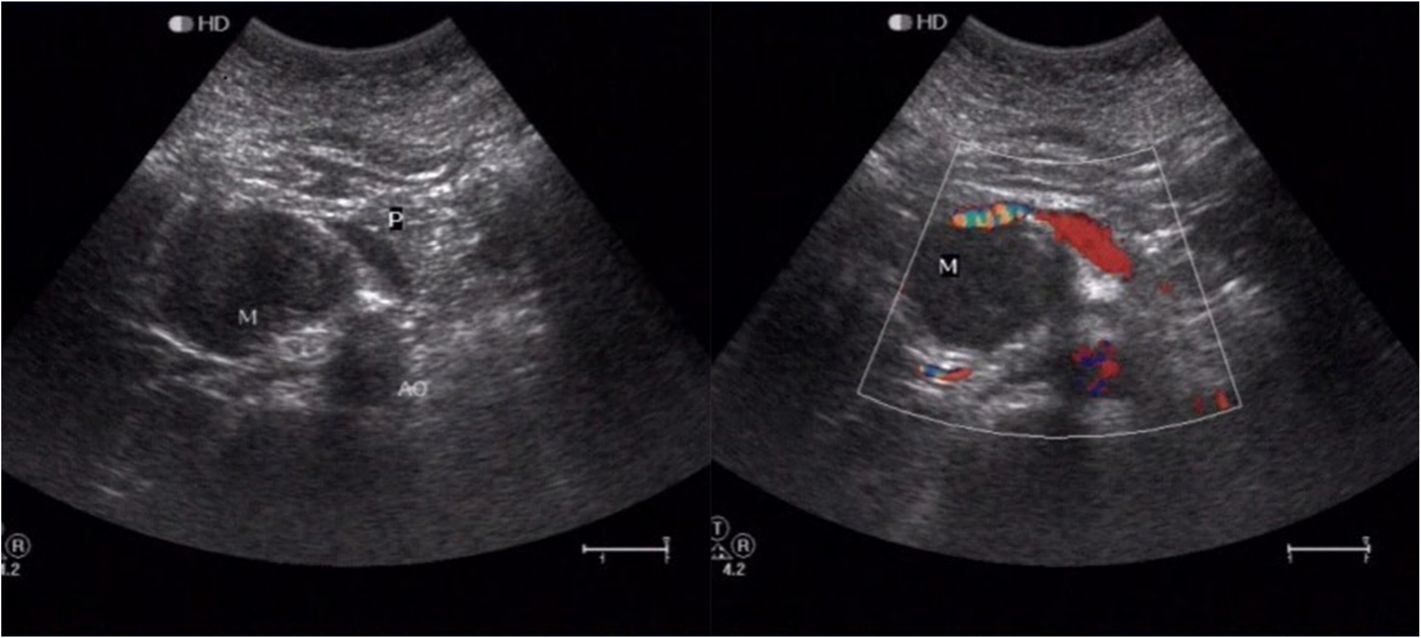
Ultrasound examination demonstrated a hypoechoic mass with irregular shaped, ill-defined and no blood signals in the retroperitoneal space with uncertain origin (P, pancreas; AO: abdominal aorta)

Initially, she was arranged to perform an upper abdominal ultrasound (US) examination. Results demonstrated a hypoechoic mass with irregular shaped, ill-defined and no blood signals in the retroperitoneal space with uncertain origin (Fig. 1), and some stones with acoustic shadowing in mild thickened wall gall bladder. Then, the CT scans with contrast enhancement was performed to identify the etiology of this mass detected on US examination. CT reports suggested a cystic SPN of pancreatic head or a retroperitoneal mass with bleeding. Finally, in order to further investigate the etiology of mass, she was recommended to perform MRI with contrast enhancement and Magnetic resonance cholangiopancreatography (MRCP).

**Fig. 2:**
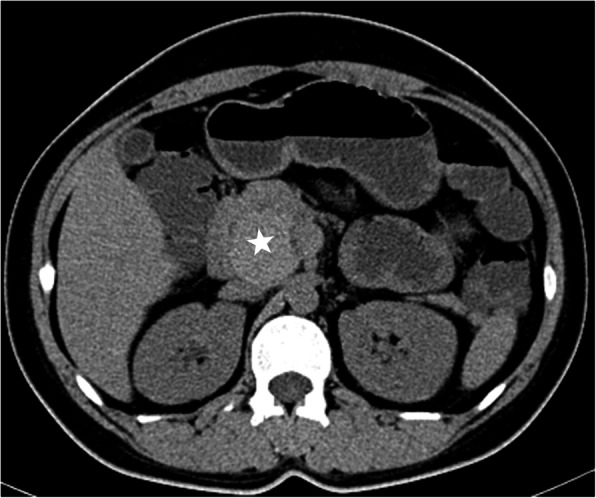
Tumor was demonstrated a lobulated, partly ill-defined lesion (star) with predominantly homogeneous hyperattenuation (mean CT value = 60HU) located on the posterior of head of pancreas on non contrast CT

**Fig. 3:**
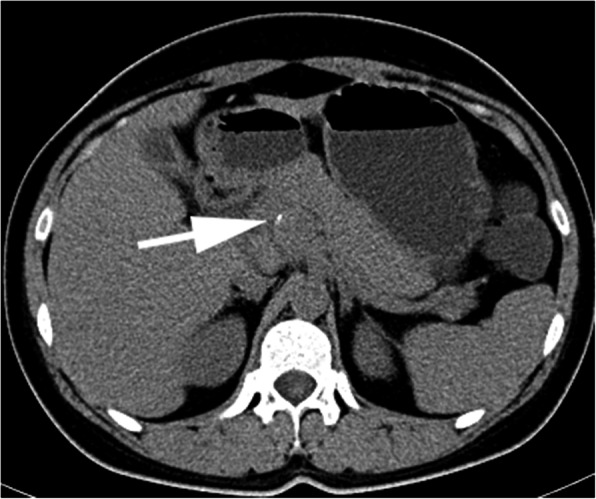
Spotted calcification was seen on the margin of lesion (arrow)

**Fig. 4:**
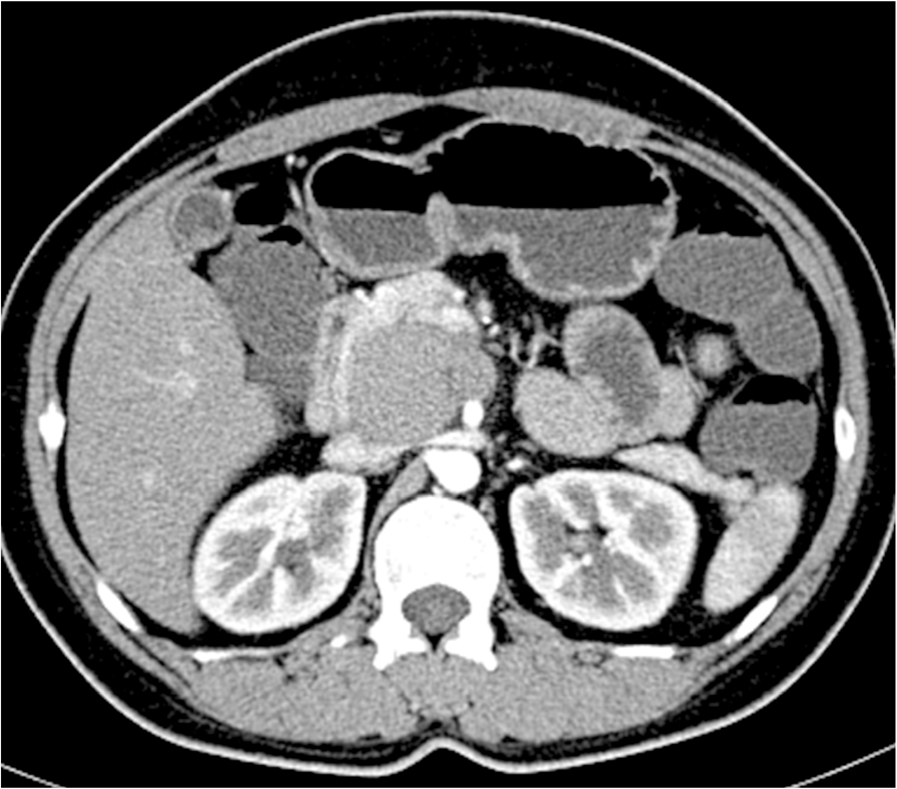
After contrast administration, the mass was manifested as a cystic lesion without obvious enhancement on artery phase

**Fig. 5:**
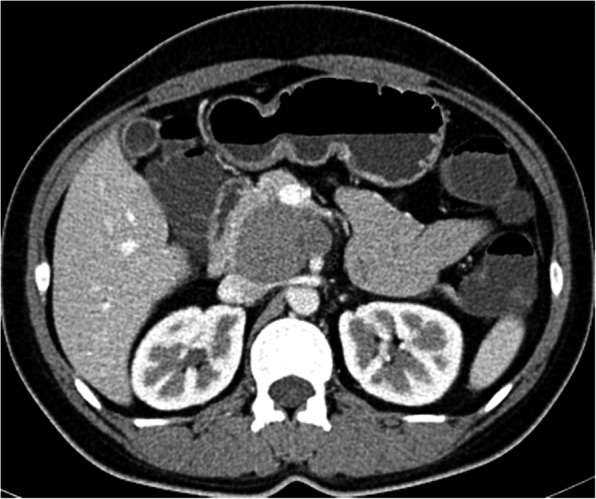
The cystic wall and thin septa of lesion was showed with mild degree of enhancement on portal phase

**Fig. 6:**
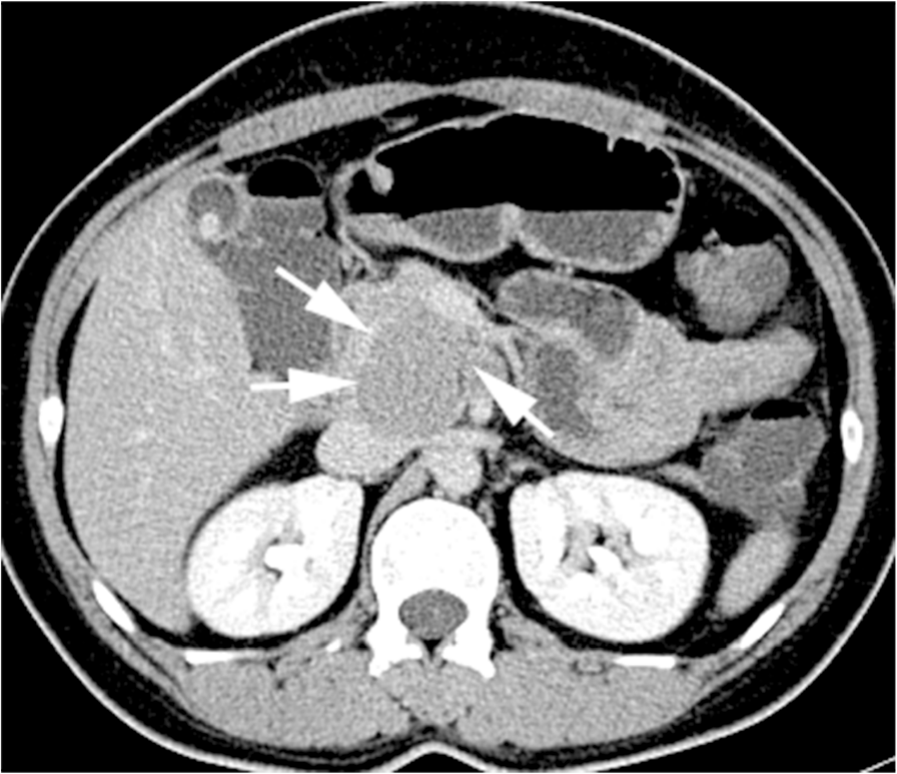
The cystic wall and thin septa was showed with moderate degree of enhancement on delayed phase (arrow)

**Fig. 7:**
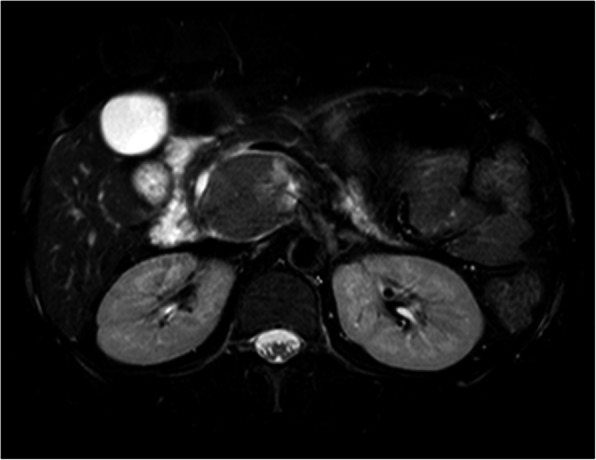
On MR, the lesion was showed predominantly slightly high signal with a small region of markedly high signal compared to the signal of normal pancreas On T2WI

**Fig. 8:**
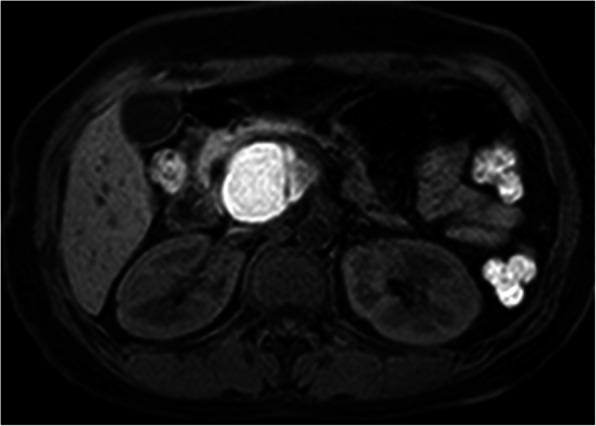
The lesion was showed high signal on FS-T1WI

**Fig. 9:**
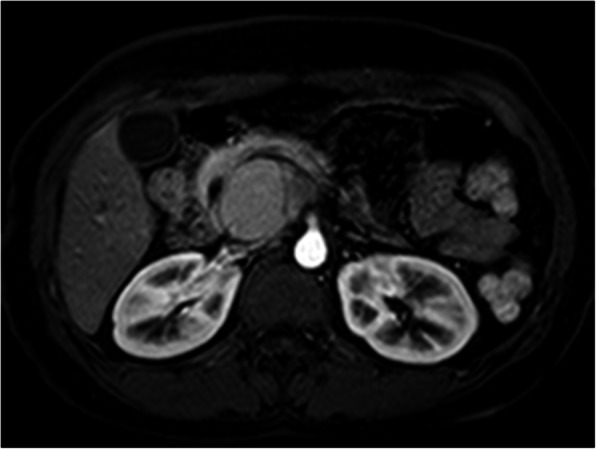
The cystic wall and thin septa of lesion was showed without obvious enhancement on artery phase

**Fig. 10:**
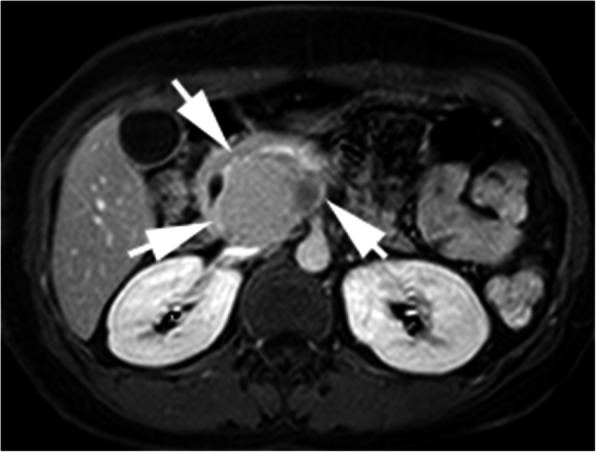
The cystic wall and thin septa was showed with moderate degree of enhancement on delayed phase (arrow), dynamic contrast enhancement pattern of lesion was the same as CT enhancement

**Fig. 11:**
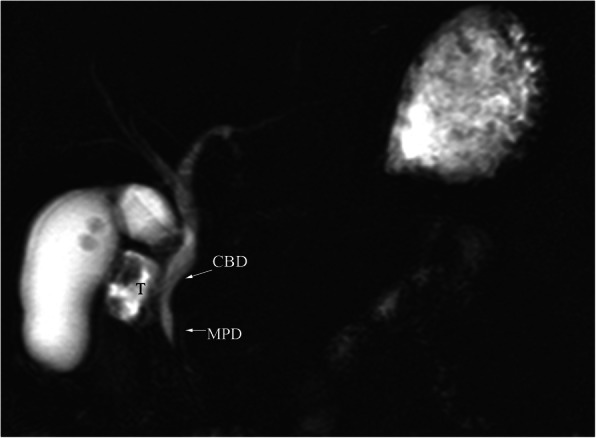
MRCP showed the communication between lesion (T) and main pancreatic duct was not seen, and the main pancreatic duct and common bile duct were not dilated

CT (SIEMENS, Sensation, 64-rows) and MR (Philips Medical systems, Achieva, 1.5 T) images showed a lobulated, partly ill-defined cystic lesion located in the pancreatic head. The maximum cross-sectional size of lesion was 3.9 cm and 5.2 cm, the maximum cross diameter of three dimensions of lesion was 5.7 cm. The cystic wall and septa was thin, even thickness, about 1.5 mm. Spotted calcification within cystic wall was found on non-contrast CT images. The lesion was demonstrated as predominantly homogeneous hyperattenuation on CT (CT value = 60HU) and homogeneous high signal without decreased signal on fat suppression sequence on T1WI, and slightly high signal compared to normal pancreas on T2WI, and hypo-intense signal on DWI (b = 1000). After contrast administration, no solid component of lesion was detected, and the cystic wall and septa of lesion was showed a gradually enhancement from mild to moderate degree over time both on CT and MR images. Lesion was adjacent to the celiac axis, splenic artery, and the portal vein. Enlarged lymph nodes in the peripheral of the lesion and the retroperitoneal space were not found. Thickened wall gall bladder with some stones but without enlargement and exudation was seen. No communication between lesion and the main duct was found on MRCP images and the main pancreatic duct and common bile duct were not dilated (Figs. 2, 3, 4, 5, 6, 7, 8, 9, 10, 11).

She was arranged a surgery after 7 days of her admission into hospital. Intraoperative findings showed the tumor was ill-defined in head of pancreas and protruded from pancreantic head to the retroperitoneum predominantly. The tumor was adhesion with the adjacent splenic artery. Partial pancreatectomy for this pancreatic head tumor and lymphadenectomy, cholecystectomy, splenic artery resection were finally performed.

**Fig. 12:**
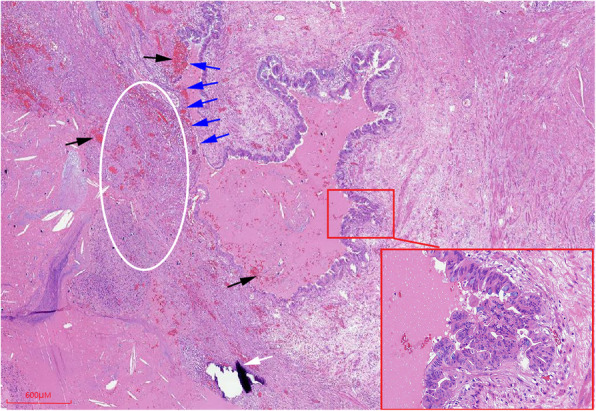
Histopathologically, intratumoral bleeding (black arrow), epithelium denudation (blue arrow), inflammatory cells (circle) under denuded area of the epithelium and focal calcification (white arrow) was seen on microphotograph (HE,× 20); region with papillary epithelium lining the cyst wall was magnified (× 200) on the bottom right corner of picture (red rectangle box)

**Fig. 13:**
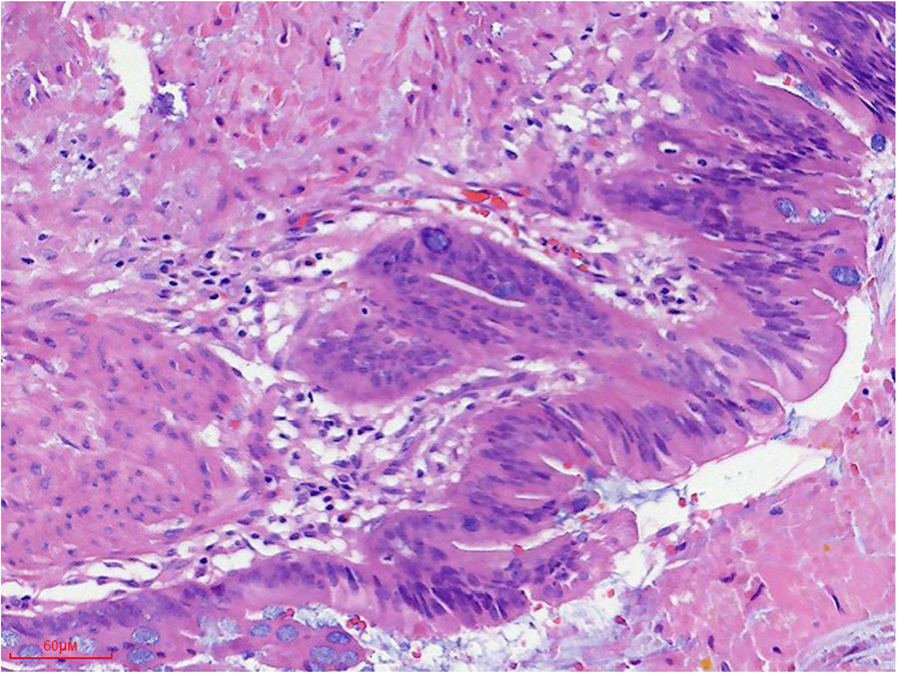
High-power view of the duct wall showing columnar mucin-containing epithelial cells and focally formed papillary structure with mild dysplasia (HE,× 200)

The lesion was pathologically confirmed as a branch type IPMN after surgical resection. Macroscopic findings of resected tumor showed that it was a cystic mass with papillary projections into the duct lumen, smooth inner lining, ill-defined, filled with dark brown liquid and some mucoid material. Microscopic findings demonstrated the inner of cystic mass most are composed of papillae lined by tall columnar mucin-producing epithelial cells, occasional goblet-type cells. The columnar epithelial cells had granular eosinophilic cytoplasm and mild degree of architectural/cytologic atypia. Some cystic wall mucosal epithelium was absent. Hemorrhage, slit-like cholesterol clefts and focal calcification were seen. The interstitium showed more acute and chronic inflammatory cells infiltration and fibrinoid exudation (Figs. 12, 13). Immunohistochemical analysis showed forinhibin-a (−), CK19 (+), CA199 (+), Ki67 (10%+).

She had a complication of pancreatic leakage after surgery, and was successfully discharged after treatments. The placement of peritoneal drainage pipe was not removed. One month latter, because of the obstrution of peritoneal drainage pipe, pancreatic leakage was recurrent and aslo successfully dismissed after a period of hospitalization. According to her follow-up imaging studies and her normal level of tumor makers, she was free from recurrence for 2 years after surgery.

## Discussion and conclusion

IPMN can complicate with acute pancreatitis (AP) [3, 4], duct perforation, fistula formation into the adjacent lumen organs (the duodenum, common bile duct, stomach, colon and small intestine) [5, 6] and spleen solid organ [7], intraductal bleeding or a massive abdominal cavity hematoma caused by rupture [1, 2], and cyst infection [8], according to previous studies. AP is the most common complications of IPMN, approximately 20% of IPMN complicated with acute pancreatitis of mild to moderate severity [4]. High-pressure caused by excessive mucin secretion in pancreatic duct is the main reason for these complications, and inflammation, auto digestion by the enzyme-rich fluids and direct invasion due to malignancy also are the possible mechanism of fistula formation [9]. The route of infection of IPMN associated with cyst infection is thought to be retrograde [10].

As we know, there are only 7 cases, including the present case, of IPMN that have been reported complicated with intraductal bleeding. The causative factors of intraductal hemorrhage may be the epithelium denudation caused by high- pressure stress, resulting in the small vessels around the epithelium injury [1]. The amount of intraductal hemorrhage always is limited, although there was a case report of a life-threatening massive abdominal cavity hematoma associated with IPMN rupture with a unknown mechanism [2].

Commonly, hemorrhage within tumor manifested as a hyper attenuation without enhancement on CT images, and a high signal on TIWI, a high or low or mixture signals on T2WI. MR imaging, especially FS-T1WI sequence, has been reported that it can more effectively detect the hemorrhage within lesions of pancreas than conventional spin-echo imaging [9, 11]. In our case, intraductal hemorrhage was showed hyper attenuation on noncontrast CT scan and high signal on FS-T1WI, consistent with the manifestation of hemorrhage in previous studies.

Interestingly, we found that the IPMN complicated with intraductal bleeding may have a locational preference for pancreatic head. In 4 of the 7 reported cases (including the present case) had the above-mentioned feature, accounting for 57.1%. And, we believed that the subtype classification of these 4 cases were BD-IPMN according to the descriptions of tumors in their studies, although there was no definitive statements about the subtype classification on their reports. This may suggests that the intraductal bleeding is relatively more frequently occurred in branch duct IPMN than main duct PMN and mixed type IPMN, with a possible underlying mechanism of a higher incidence of more serious pressure overload caused by massive mucin production resulting in more easier and serious of epithelium denudation, and more frequent the small vessels injury, since the high-pressure within branch duct in the pancreatic head could not easily transmit to the main duct, especially for cases of branch duct IPMN with no obvious communication to the main duct.

Calcification within IPMN was not commonly seen. The incidence of detection of calcification was approximately 20%, according to the Perez-Johnston’s study in a large cohort of 164 IPMNs [8]. The shape of calcification can be classified as spotted or punctate, coarse and eggshell on CT images. The spotted or punctate calcification is the most common seen in IPMN and has not been associated with malignancy, but the coarse calcification may be a worrisome feature of malignancy [8]. Calcium salt deposits facilitated by the thick mucin and coexisting chronic calcifying pancreatitis may be the main reasons of presence of calcification within IPMN [12, 13].

In our case, what extreamly rare is that the synchronous presence of calcification and intraductal bleeding within IPMN in a young woman were detected on CT and MR images. Considering the gender, age, calcification, intratumoral bleeding, gradually enhancement from mild to moderate degree of cystic wall and no obvious communication to the normal main duct, our case of IPMN mimicked a cystic SPN, and that is the reason for a preoperative misdiagnosis as a SPN.

Therefore, we propose that IPMN may need to be taken into account in the differential diagnosis when pancreatic cystic lesions occur in young women with bleeding, calcification, progressive enhancement of cystic wall and no communication with the main pancreatic duct.

## Data Availability

Not applicable.

## Abbreviations

IPMN: Intraductal papillary mucinous neoplasm
SPN: Solid pseudo-papillary neoplasm
LMP: Last menstrual period
BMI: Body mass index
US: Ultrasound
CT: Computed tomography
MRI: Magnetic resonance imaging
MRCP: Magnetic resonance cholangiopancreatography
T1WI/ T2WI: T1/T2 weighted image
FS T1WI: Fat suppressed T1 weighted image
DWI: Diffusion weighted imaging
AP: Acute pancreatitis
(CA199): carbohydrate antigen 19–9
CEA: Carcinoembryonic antigen
AFP: Alpha-fetoprotein
